# Novel combinatory method for surface and crystallinity analysis of crystalline materials: Combinatory analysis method for crystalline materials surface

**DOI:** 10.1016/j.mex.2023.102105

**Published:** 2023-03-07

**Authors:** Vladyslav Matkivskyi, Arne Karstein Røyset, Gaute Stokkan, Pål Tetlie, Marisa Di Sabatino, Gabriella Tranell

**Affiliations:** a NTNU, Alfred Getz' vei 2B, Trondheim 7034, Norway; b SINTEF, Alfred Getz' vei 2B, Trondheim 7034, Norway; c SINTEF, Høgskoleringen 5, Trondheim 7034, Norway

**Keywords:** C-Si chemical etching, C-Si surface analysis, Crystallographic mapping, EBSD alternative, White light interferometry, Laue tool, Multi-crystalline silicon, Texturing, Combinatory analysis method for crystalline materials surface

## Abstract

This work is dedicated to developing a method of combined surface morphology- and crystallographic analysis for crystalline silicon. To demonstrate the applicability of the method, a series of chemical operations, such as polishing and texturing, were applied to multi-crystalline silicon samples. The samples were pre- and post-analysed with WLI and Laue techniques, and the experimental data allowed construction of maps for crystal orientation to etching rate dependency. The study illustrates the strengths of the combinatory technique as an alternative to existing techniques such as atom force microscopy (AFM) and electron backscatter diffraction (EBSD).

• Combination of LAUE tool and white light interferometry techniques.
• Alternative time-effective method to EBSD.
• Analysis of surface morphology and crystallographic properties for chemical processing.

## Graphical abstract

**Figure:**
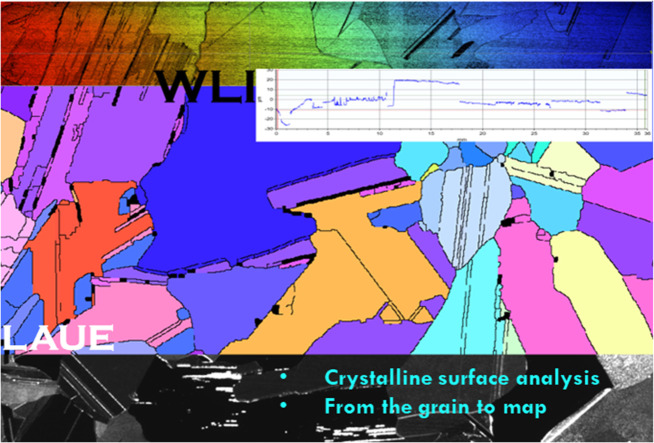

Specifications table

**Table:** 

Subject area:	Materials Science
More specific subject area:	*Surface analysis*
Name of your method:	Combinatory analysis method for crystalline materials surface
Name and reference of original method:	*Novel combinatory method for surface and crystallinity analysis of crystalline materials*
Resource availability:	*White light interferometry equipment (Bruker), Laue tool, OIM 64, Vision 64, MTEX.*

## Background and state of the art

The modern semiconductor silicon industry and research domains connected to this industry use a wide range of surface analysis techniques to study crystal structure, -orientation, -morphology, -grain structure and -2d defects (grain boundary, twins, etc.). Common techniques include scanning electron microscopy (SEM) [1], atomic force microscopy (AFM) [2,3] and electron backscatter diffraction (EBSD) [4]. The crystalline silicon surface properties and crystallography are important as they largely affect the efficiency of solar cell processing operations such as texturing, polishing and etching conducted on the surface.

Scanning techniques such as stylus profilometry [3] and AFM are important techniques for “direct” surface scanning. Stylus profilometry is one of the widely used instruments for surface morphology measurements because of its simple working principle which is based on the conversion of a mechanical response to electrical signal. This technique can be used for relatively large sample areas up to 300 mm. However, the lateral resolution of this technique is limited to maximum 50 nm. In the modern silicon industry, morphological processing on Si has a nano-meter scale and to observe morphological changes in porosity [5], nano-columns [6] etc., more precise techniques are hence required.

AFM is a surface analysis technique which is based on van der Waals forces [3]. The lateral resolution of this technique is limited by the maximum curvature of the AFM tip, although it allows observing the morphology on the dozens of nm scale [7]. Additionally, conductive AFM can be used to observe electrical properties such as conductivity and electrical recombination of extended defects such as grain boundaries [8] and dislocations [9].

EBSD is a powerful technique mainly used for analysis of crystalline materials [4]. This technique is developed in a SEM system and uses high resolution digital EBSD detector [3]. The main principle of EBSD is based on the use of transmission Kikuchi diffraction (TKD) [4]. Structural material properties such as grain size, misorientation angles, grain boundaries and micro-texture might be derived from the EBSD measurement analysis.

The main drawback for most surface scanning techniques as described above is the processing time. Additionally, the EBSD technique has the inherent limitations of the SEM, i.e. the area of analysis is small and hence, it is time consuming to complete recognition and indexing of different crystalline patterns on a larger wafer area [10].

The AFM technique has almost the same challenges as the EBSD. However, use of the AFM does not require a vacuum environment. The area of the scan is larger than the EBSD. One of the main drawbacks of this technique is the scale on which the analysis can be carried out, i.e. AFM scanning is typically conducted on the micron to millimetre scales. Thus, larger area scales are both time-consuming and less precise for AFM analysis [7].

Within this context, a combinatory method for overcoming these obstacles when applied to surface analysis of multi-crystalline Si wafers, was developed. As an alternative to the SEM EBSD technique, the Laue scanner was developed. It allows wide area scanning and is, like EBSD, based on X-ray diffraction [11]. The method combines the use of Laue scanning and White Light Interferometry (WLI)[12], and allows to correlate the crystal orientation of each grain of a silicon wafer to its etching rate.

## Method details

### Laue tool scanning

The first of the two techniques combined in this study is Laue scanning. This instrument was implemented as large area alternative to EBSD scanning for obtaining the crystal orientation of individual crystals on a mc-Si wafer surface.

The working principle of the Laue tool is based on X-ray beam scanning. To obtain a scanned sample map, the Laue tool takes a series of optical images of the sample to identify possible grain contours and create a map for the subsequent X-ray scanning. The crystals on the surface are represented by a colour where the colour represents the change in crystal orientation on the sample. During the scanning process, a backscatter diffraction pattern of every lattice plane on the sample surface is created by the X-ray. By wavelength and lattice spacing matching at the interface, according to Bragg's law (Eq. 1), all crystal orientations are measured. Further, these orientations are expressed by the Euler's angles (rotation of crystal) and coordinate system of the sample. As a result, the Laue tool generates a file with sample surface coordinates and their corresponding Euler's angles [11].

(Eq. 1) n×λ=2d×sinθ

To determine the crystal orientation obtained from the Laue scan, the data needs to be converted to the EBSD format using Bunge Euler angles. The data may be analysed by, for example, the TSL OIM (orientation mapping microscopy) software, where an hkl colour-corresponding map is created.

The Laue scanner allows crystal orientation measurements for both as-cut as well as polished samples. Chemical polishing of the wafer improves the surface refractive index and hence makes the optical identification of a grain boundaries more precise.

First, the sample is placed onto the stage (Fig. 2). Second, the Laue stage is moved in the Z-direction to set an optimal distance between scanner and sample. The scanning procedure of the Laue tool can be divided into 2 modes, which are i) optical and ii) X-ray (Laue) scan. During the optical mode scanning, the sample surface is illuminated with the white light and scanned by the built-in microscope, in order to distinguish different grains on the sample. All detected crystal grains are subsequently scanned by the X-ray, to obtain backscatter diffraction of the present lattices. As a result, all crystal orientations are measured and the hkl colour map of the sample is created.

**Fig. 1:**
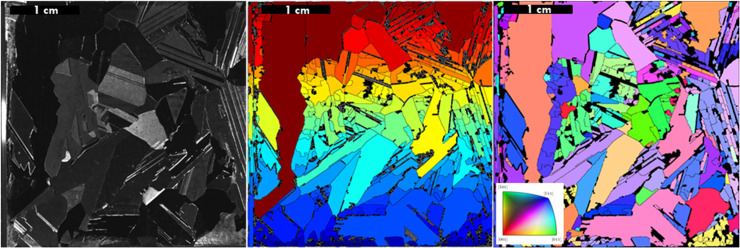
Images obtained by scanning with Laue tool of a multi-crystalline Si wafer.

**Fig. 2:**
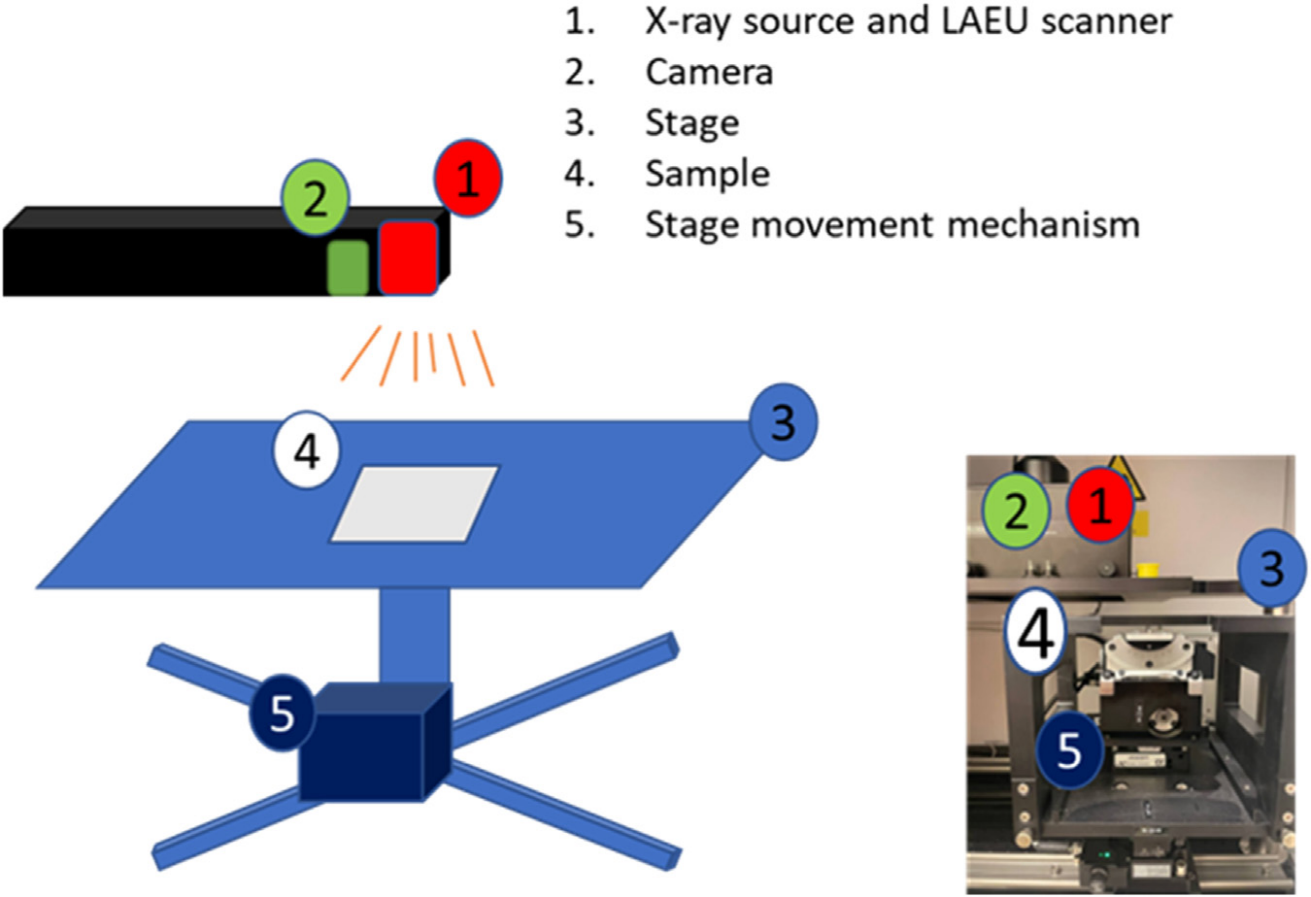
LAUE tool schematic with real view.

### White light interferometry scanning

**Fig. 3:**
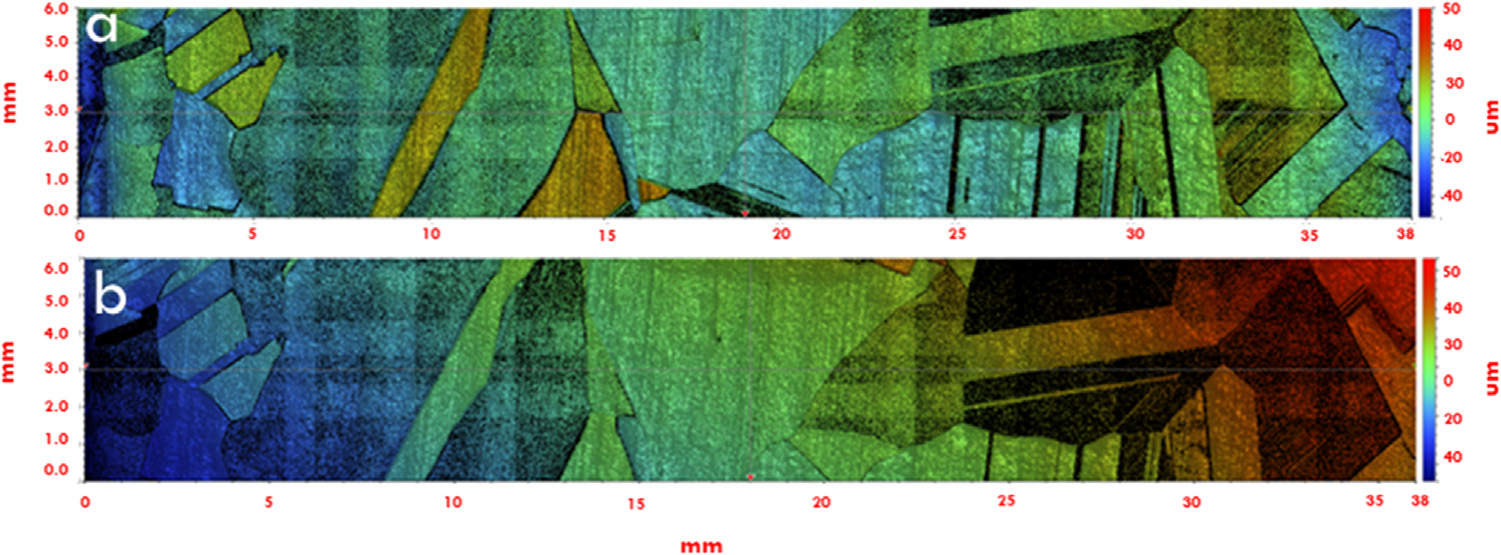
Example of the white light interferometry scan for the multi-crystalline Si surface before (a) and after etching operation (b).

White light interferometry (WLI) is the complementary method used for quantitative determination of the surface roughness of the etched c-Si wafer. The working principle of WLI is based on light reflectance from a specimen surface. Received back from surface reflected light is synthesized into surface shape, with the contrasts in reflectance being an indication of the different sample surface areas (crystals) height variations. Thus, the WLI analysis allows distinguishing height differences on a wider wafer area than the AFM, i.e. it is more similar to the resolution in optical microscopy. Additionally, the WLI technique gives the possibility of obtaining 2D and 3D topography of the specimen, while having a lateral resolution down to 100 nm scale. The main advantages of WLI are: i) contactless and ii) non-destructive analysis, which eliminates any risk of damaging and contaminating the sample or its surface. A detailed map of a multi-crystalline Si wafer in the etched and non-etched conditions scanned with the WLI is presented in Fig. 3.

The scanning process by the WLI consists of light beam emission onto the Mirau interferometer [12] and the surface of the specimen. The interface fringes created by the combined reflected beams are analysed by a charge-coupled device (CCD) camera [12]. A detailed scanned image of the surface height along the scanning axes is obtained by constructing fringes along the scanning range. Moreover, a cross-action mode might be useful and precise for a determination of the height difference between grains on a sample.

The WLI tool includes such techniques as diffraction grating, coherence probe, white light scatter plate interferometry combined with modern software and calculation efficiency [13]. This makes WLI a powerful tool for various applications. Our work presents the use of WLI as a suitable and time effective tool for the wide area profile scanning of Si wafers with high accuracy determination on the micro scale. WLI analysis for measuring Si-wafer surface profile can be conducted in two modes. The first mode allows obtaining a colour (surface height) representation of the depth profile for each small, scanned sample surface area. The second mode is a so called “stitching” mode which allows stitching multiple scans together, creating a large area scanned profile. During the sample analysis by WLI, the maximum scanning area was 6 × 36 mm. Using a wider area scan will affect the quality of the image and cause problems in the stitching process.

The surface height of a certain point on the specimen is first aligned with the stage (or sample holder), thus allowing to set a zero level for the height measurement on the sample. This can be used to obtain a precise area height difference between, for example, an etched and unetched sample area. Example of such measurement is presented in the Fig. 3.

Fig. 3 illustrates a scanned sample with WLI technique. Depending on the height difference on the surface morphology, it is marked with colour. To the right of the figure is a colour scale which allows determining height difference on the pointed area with respect to the “zero” level. The obtained measurements were analysed in Vision 64 software, where area height profile can be estimated.

## Method validation

The two tools described above were combined to determine the etching response of individual crystals in multi-crystalline Si wafers to different etching solutions. The method consisted of the main experimental steps: wafer polishing, Laue tool scanning, WLI scanning, wet chemical etching, second round WLI scanning as illustrated in Fig. 3.

### Sample preparation (polishing of the Si surface)

To prepare the as-cut mc-Si wafer samples, chemical cleaning and subsequent polishing are required. The multi-crystalline Si wafers were laser cut into 4 × 4 cm^2^ pieces and cleaned in the RCA1 solution at 65-70°C and dried. Two subsequent wet chemical polishing treatments of the sample were carried out as presented in the Table 1.

**Table 1:** Cleaning and polishing solutions of the investigated wafers.

*Solution 1* *(polishing)*	*Wt%*	*Solution 2 (polishing)*	*Wt%*	*RCA 1 solution* *(cleaning)*	*Wt%*
*HF (49%)*	*<10*	*KOH*	*<6*	*DI water*	*71*
*HNO_3_ (75%)*	*50*	*NaOCl*	*<2*	*NH_4_OH*	*14*
*CH_3_COOH*	*40*	*IPA*	*<5*	*H_2_O_2_*	*14*
		*Di water*	*80*		

### WLI and Laue Scanning

**Fig. 4:**
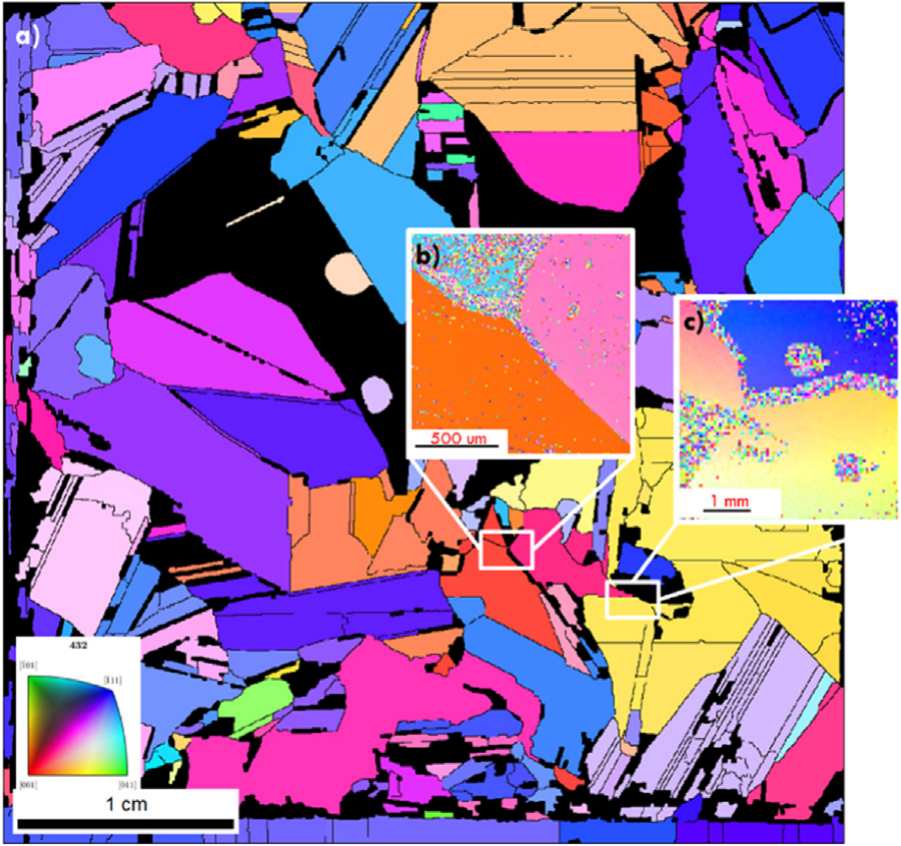
(a) Laue tool crystallographic map of a sample; (b) (с) EBSD scanned and indexed areas of the same sample.

The combination of white light interferometry and Laue scanning techniques has been successfully applied as a research method, as described in more detail in our work [13], where determination of the etching rate in several different etching solutions for different crystal orientations on multi-crystalline wafers, was conducted. Following the procedures outlined in section 2, Laue scanning was first conducted on the specimen. Additionally, a few areas of the same sample were scanned by EBSD to confirm the results of the Laue scanning. Fig. 4 illustrates the Laue (a) and EBSD (b,c) scans.

As illustrated by Fig. 4, the orientational indexing for Laue and EBSD scanning are identical. However, obtaining a 4 × 4 cm^2^ area scan by the EBSD technique is very time consuming (∼17 s/pattern) compared to the Laue technique (∼6 s/pattern). Thus, Laue scanning is almost three times faster than SEM EBSD. Fig. 4 combines a full sample Laue scan (a) with two area scans of the same sample (b,c) by EBSD.

**Fig. 5:**
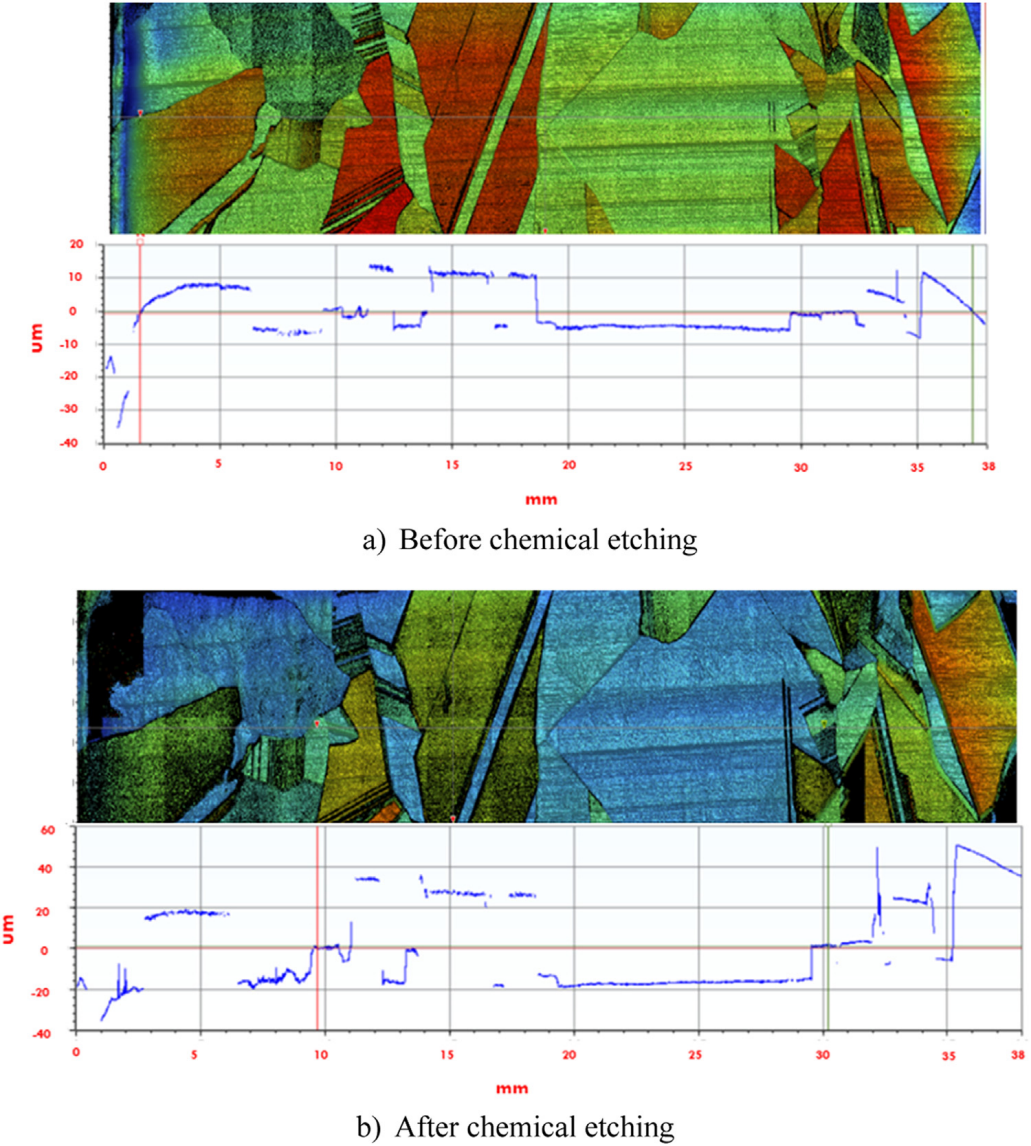
White light interferometry scans of the sample with additional levelling details of the surface (X – sample scan length, Y – surface height profile). Colour map corresponds to the same type as on the fig. 3.

After orientational indexing of the scanned sample, the WLI scanning was applied to the sample. Fig. 5 (a) demonstrates a 2D area and surface profile WLI scanned sample before the etching operation, respectively. Fig. 5 (b) shows the same sample scan of the etched sample. Thus, by comparing the two surface profiles using the same “zero” level, it is possible to estimate the height difference between unetched and etched profiles. Using this information, the etching speed for individual crystal (orientations) was calculated.

**Fig. 6:**
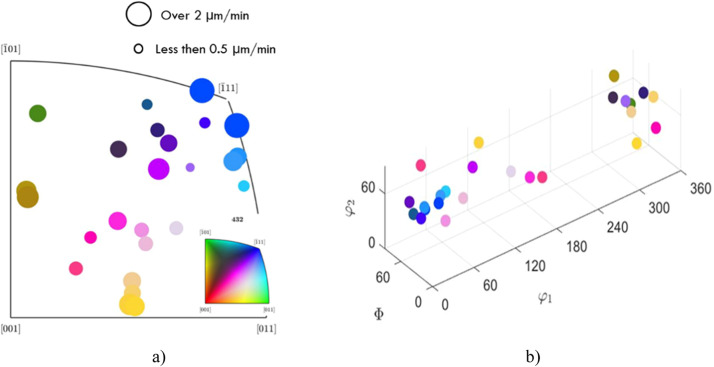
Examples of MTEX graphical maps for (a) etching speed to grain orientation and (b) grain orientation to placement in the Rodriques space (φ1 and φ2 – rotation angles).

Following the combination of the WLI and Laue measurements, an etching speed map can be generated for the grain orientations on the sample, using the Matlab MTEX toolbox software. This is possible by insertion of previously calculated etching rates (by WLI) into MTEX algorithm. Fig. 6 demonstrates such plots, where (a) presents a 2D colour-map diagram where previously indexed sample grains correspond to the same colour of the circle located in the three main crystallographic orientations, plotted in 2D space. The size of the dots is corresponding to the calculated etching rate for this orientation. Thus, it is possible to distinguish trends regarding grain orientation and etching speed. Fig. 6 (b) represents different grain orientations in axis angle (Rodrigues Space). Thus, it is possible to see 3D space position of the certain grain orientations. Further, analysis of such plots is presented elsewhere [14].

## Summary

The combination of Laue and WLI scanning allows obtaining a large data set for c-Si surface analysis. The developed method provides a full area crystallographic scan of multi-crystalline Si wafers. Additional use of the WLI allows construction of crystallographic surface maps, estimating surface roughness of a large area. The effect of surface treatments such as polishing, texturing, reactive ion etching, metal assisted chemical etching etc can be quantified. Using a combination of this data and a software tool such as MTEX, it is possible to quantify the effect of the surface treatment (specifically the etching rate) on the c-Si surface grain orientation. Furthermore, white-light interferometry can also be applied as a stand-alone technique for characterization of texture-related features in mono-crystalline wafer processing.

## Declaration of Competing Interest

The authors declare that they have no known competing financial interests or personal relationships that could have appeared to influence the work reported in this paper.

## Data Availability

Data will be made available on request.
